# Validation of the German version of the STarT-MSK-Tool: A cohort study with patients from physiotherapy clinics

**DOI:** 10.1371/journal.pone.0269694

**Published:** 2022-07-01

**Authors:** Sven Karstens, Jochen Zebisch, Johannes Wey, Roger Hilfiker, Jonathan C. Hill

**Affiliations:** 1 Department of Computer Science, Therapeutic Sciences, Trier University of Applied Sciences, Trier, Germany; 2 Physio Meets Science, Leimen, Germany; 3 Department of Computer Science, Formerly Therapeutic Sciences, Trier University of Applied Sciences, Trier, Germany; 4 School of Health Sciences, HES-SO Valais-Wallis, Leukerbad, Switzerland; 5 School of Medicine, Keele University, Staffordshire, United Kingdom; Mugla Sitki Kocman Universitesi, TURKEY

## Abstract

**Background:**

The STarT-MSK-Tool is an adaptation of the well established STarT-Back-Tool, used to risk-stratify patients with a wider range of musculoskeletal presentations.

**Objective:**

To formally translate and cross-culturally adapt the Keele STarT-MSK risk stratification tool into German (STarT-MSK_G_) and to establish its reliability and validity.

**Methods:**

A formal, multi-step, forward and backward translation approach was used. To assess validity patients aged ≥18 years, with acute, subacute or chronic musculoskeletal presentations in the lumbar spine, hip, knee, shoulder, or neck were included. The prospective cohort was used with initial data collected electronically at the point-of-consultation. Retest and 6-month follow-up questionnaires were sent by email. Test-retest reliability, construct validity, discriminative ability, predictive ability and floor or ceiling effects were analysed using intraclass correlation coefficient, and comparisons with a reference standard (Orebro-Musculoskeletal-Pain-Questionnaire: OMPQ) using correlations, ROC-curves and regression models.

**Results:**

The participants’ (n = 287) mean age was 47 (SD = 15.8) years, 51% were female, with 48.8% at low, 43.6% at medium, and 7.7% at high risk. With ICC = 0.75 (95% CI 0.69; 0.81) test-retest-reliability was good. Construct validity was good with correlations for the STarT-MSK_G_-Tool against the OMPQ-Tool of r_s_ = 0.74 (95% CI 0.68, 0.79). The ability of the tool [comparison OMPQ] to predict 6-month pain and disability was acceptable with AUC = 0.77 (95% CI 0.71, 0.83) [OMPQ = 0.74] and 0.76 (95% CI 0.69, 0.82) [OMPQ = 0.72] respectively. However, the explained variance (linear/logistic regression) for predicting 6-month pain (21% [OMPQ = 17%]/logistic = 29%) and disability (linear = 20%:[OMPQ = 19%]/logistic = 26%), whilst being comparable to the existing OMPQ reference standard, fell short of the a priori target of ≥30%.

**Conclusions:**

The German version of the STarT-MSK-Tool is a valid instrument for use across multiple musculoskeletal conditions and is availabe for use in clinical practice. Comparison with the OMPQ suggests it is a good alternative.

## Introduction

Musculoskeletal (MSK) disorders comprising pain in the region of the lower back, the neck, or osteoarthritis affecting the joints of the upper or lower extremities are among the leading causes of disability. These complaints often have a chronic course and their burden on individuals and society is large [1–3]. Due to aging populations, it is estimated that the prevalence of MSK conditions will further rise [4]. Typically, the majority of patients with these conditions are managed by general practitioners and physiotherapists [5]. Patient-reported measurement instruments are used by these clinicians to a varying degree, but there is a need for generic prognostic tools and risk stratification methods that are usable across a variety of body sites to facilitate targeted treatment decision-making [6, 7]. A back-specific instrument, specifically designed to establish the prognosis of patients in primary care is the Keele STarT-Back-Tool (**S**ubgrouping for **Tar**geted **T**reatment). It allocates patients to one of three prognostic sub-groups (low, medium and high risk) in which they receive a risk-matched treatment [8–10]. This procedure has shown effectiveness and has been implemented in routine care in the UK [9, 11]. Internationally, the successful reproduction of the research results remains limited, although practitioners describe positive experiences in clinical practice [12, 13]. One criticism is that this tool is limited to patients with low back pain and a tool applicable to a broader group of musculoskeletal patients would have much great appeal and be easier to implement [14].

Through a programme of research the STarT-Back-Tool has therefore been adapted and validated to produce the Keele STarT-MSK risk stratification tool (STarT-MSK) for use in a broader musculskeletal patient population [15]. This approach has been supported by a recent umbrella review indicating that there are a number of common prognostic factors among patients with MSK-complaints including: worse baseline function, higher symptom/pain severity, worse mental well‐being, more comorbidities, older age and higher body mass index [16]. Several translations of the STarT-MSK-Tool are available, but a German version did not exist and knowledge about its measurement properties is limited [17–21].

A translated version of the original STarT-MSK could support German physiotherapists, physicians or other health professionals to be able to provide risk-based stratified care for musculoskeletal disorders [14, 18]. Risk-based stratified care may help clinicians to better target treatments according to a patients’ individual risk status, thereby maximising the benefits of care and reducing unnecessary treatments and costs [22, 23]. Consensus on the primary care management options relevant for each risk-group has been identified for UK primary care [24], but this may need to be adapted to the German context. Sowden et al. described various matched treatments for back pain ranging from one-off advice sessions for low risk patients to more comprehensive solutions addressing patients with complex biopsychosocial prognostic factors for high risk patients [25]. To develop comparable procedures, recommendations for matched treatments were gathered for patients with MSK-conditions [24, 26, 27], and together with the tool were integrated by an international research group developing a web-app informing first contact clinicians in their clinical decision making [28].

The objective of this study was, to formally translate and cross-culturally adapt the STarT-MSK-Tool into German (German version: STarT-MSK_G_). Moreover, we aimed to investigate its test-retest reliability, construct validity discriminative ability, predictive ability and floor or ceiling effects.

## Methods

### Design

A cohort study including a retest (t1) and a half-year follow-up (t2) in addition to the intitial assessment (t0) was conducted. Patients were recruited from physiotherapy clinics (n = 7). The inclusion criteria were patients 18 years or older with acute, subacute or chronic complaints in the region of the lumbar spine, the hip or knee, the shoulder or the neck. The exclusion criteria were those with a known or suspected tumor, an acute inflammatory condition, recent musculoskeletal-related surgery (last six months) or trauma (last 3 months). German language skills had to be sufficient to complete the study questionnaires and participants had to provide written consent and their email and telephone details for follow-up purposes.

Initial data (t0) was collected electronically in the clinics via SoSci Survey [29]. The invitations to answer the t1- and t2-questionnaires were sent by email. To counter memory effects and at the same time minimize changes due to the natural course, a period of one week between t0 and t1 was aimed for [30]. To reduce drop-outs, patients who did not respond to a t1- or t2- invitation received a reminder after one week and were phoned after two weeks.

Ethical approval was granted by the Ethics Committee of Trier University of Applied Sciences, Computer Science/Therapeutic Sciences (registration ID: 01–2019). All patients gave their written informed consent for participation before enrollment in the clinics.

### Translation and cross-cultural adaptation of the STarT-MSK-tool

The instrument validated in this study is the STarT-MSK_G_. There are two versions of the STarT-MSK-Tool: (1st) a self-report version which was used in this study and (2nd) a clinical interview version. A copy of the instrument can be requested here: www.keele.ac.uk/startmsk.

The translation and cross-cultural adaptation was done according to internationally accepted guidelines and with permission for translation from the developers of the original version [31]. The translation committee consisted of three people (SK, JW, JCH). Of those two had extensive experience in cross-cultural adaptation [32, 33]. A coordinator collected and synthesized translations. Forward translations were carried out by three people with German mother tongue; one lay person and two physiotherapists. Two of these translators were German, and one of the physiotherapists was from Switzerland to facilitate cross-national validity. The three forward translations were synthesized into a final forward version by the coordinator. This version was sent back to the translators and comments were invited. The backward translations were done by two non-medical translators who were native speakers of English. The two backward translations were sent for discussion to the developers of the original English version. Very good conformity of the backward translations with the original version was shown, but item ten was revised changing ‘pains’ (‘Schmerzen’) to ‘pain condition’ (‘Schmerzproblematik’).

To check for acceptability and comprehension a pre-test was carried out with 10 patients from a German physiotherapy clinic. A Think-Aloud method was utilized, while the tool was completed [34]. Moreover, patients were asked open questions to determine if they experienced any problems with the tool. Due to grammatical reasons the German version of item eight begins with the time frame (‘the last two weeks’) and ‘feeling down/depressed’ follows. This was preferred by the participants of the pretests, after two alternatives were presented. A report describing the translation process and including the different translations was sent to the developers and the German version was confirmed. A copy of the German version can be requested here: www.keele.ac.uk/startmsk.

### Reference instruments

To test for construct validity several reference instruments were added. Based on a formative model, both, the STarT-MSK like the OMPQ assess the risk for future pain and disability, using a set of items of known biopsychosocial risk factors [15, 35, 36]. Moreover, depending on the patients’ complaints one of the following instruments were used to determine disability: German version of the Neck Disability Index (NDI) [37], Shoulder Pain and Disability Index (subscale disability, SPADI_DIS_) [38], Roland Morris Disability Questionnaire (RMDQ) [39] or Western Ontario and McMaster Universities Osteoarthritis Index (subscale disability, WOMAC_DIS_) [40]. Pain intensity was measured using the mean of three eleven-point box-scales for least, average (over the previous two weeks), and current pain [41, 42].

The STarT-MSK comprises of 10 items. The first item is an 11-point numeric pain rating scale. The other nine items have a dichotomous response option: yes/no. To calculate a sum-score, the items are recoded (item 1: 0–4 = 0 points, 5–6 = 1 point, 7–8 = 2 points, 9–10 = 3 points; item 2 to 9: yes = 1 point, no = 0 points). The final score is calculated by summarizing the point for all 10 items, with a possible total score ranging from 0 to 12. Based on cut-off points established for the original version, a total score of ≤4 points indicates low risk, a total score between 5–8 points medium risk, and ≥9 points high risk for persisting pain disability [15].

To determine the OMPQ score the sum of the five subscale means was computed resulting in a possible range from 0 to 50 points [35]. The RMDQ-score equals the number of the items checked positive by the patients and can range from 0 to 24 points [39]. To the NDI-score, each question adds 0 to 5 points to a total maximal sum-score of 50 which is transformed to percentages ranging from 0 to 100 [37]. The WOMAC_DIS_-score was calculated by summarizing the item values, then divided by the number of items resulting in a range from 0 to 10 points [40]. The SPADI_DIS_-score was calculated by summarizing the item values, then divided by the number of valid items, with maximally one non-valid item accepted. This also resulted in a score ranging from 0 to 10 points [38].

### Statistical analyses

**Descriptive statistics** were calculated to characterize the study population and each subgroup. The baseline characteristics of the study participants are provided to allow interpretability of the study sample. Moreover, numbers on recruitment rate, drop-outs and missing data were described.

To investigate the **test-retest reliability** the intraclass correlation coefficient (ICC based on a two-way random effect, absolute agreement model (2.1)) was used. *An ICC above 0*.*50 was considered acceptable* [43]. Additionaly, Cohen’s Kappa for agreement on item level was calculated to further explain test-retest-reliability.

For convergent **construct validity** the STarT-MSK_G_ was related to the OMPQ. Spearman correlations were calculated for the time point t0. A priori a positive correlation was expected, with higher scores meaning worse prognosis on both instruments. The magnitude of the reported correlation coefficient was evaluated with a correlation of 0.1–0.3 considered to be small, >0.3–0.5 to be moderate, and greater than 0.5 to be large [44]. *At least a moderate correlation of greater than 0*.*4 was considered sufficient*. Additionally, to visually represent the correlation of the instruments, box and whisker plot graphs were be presented using the OMPQ-score for each subgroup defined by the STarT-MSK_G_ score. Next to the relation with the OMPQ, coefficients (Spearman) for the correlation with the reference instruments for disability were calculated across the pain sites (NDI, RMDQ, WOMAC_Dis_, SPADI_Dis_. In comparison to the OMPQ lower correlations were expected.

**Floor and ceiling effects** were considered present if more than 15% of the responders achieved the lowest or highest possible score [45]. *It was expected that ≤ 15% of the responders would achieve the lowest or highest possible score*.

To assess STarT-MSK_G_’s **discriminative ability** ROC (receiver operating characteristic) curves with areas under the curves (AUC) and 95% confidence interval (CI) were computed [46]. The curves were calculated for poor physical status at t0 (RMDQ [39], NDI [37], WOMAC_DIS_ [40], SPADI_DIS_ [38]). Moreover, ROC curves with AUC were computed for pain intensity and disability for all patients. To determine if a patient was a ‘case’ on reference standard instruments, the individual’s scores were compared to cut-off values defined in the literature: RMDQ ≥ 4 [47], NDI ≥ 15 [48], WOMAC_DIS_ ≥ 2.1 [49], SPADI_DIS_ ≥ 4.1 [50].

Adjectives that can be used to describe AUC-values have been proposed by Hosmer and Lemeshow with an AUC = 0.5 suggesting ‘no discrimination’, 0.7 to < 0.8 considered ‘acceptable discrimination’, 0.8 to 0.9 considered ‘excellent discrimination’ and >0.9 considered ‘outstanding discrimination’ [51]. *At least acceptable discrimination for disability was expected*.

To analyse the **predictive ability** the t0 score of the STarT-MSK_G_ **was used** as the predictor variable in univariate linear regression. *It was aimed to explain a proportion of at least 30% of variance in the outcome (disability and pain)*. For comparison purposes the variance explained by the OMPQ was also calculated. Additionally, logistic regression analyses were performed. For dichotomization of disability the thresholds given above were used (see discriminative ability), for pain intensity the median was used (with 2.7 at t0 and 4.3 at t2 this fitted well with thresholds described in the literature [52, 53]). The R^2^-statistics (adjusted/ Nagelkerke) explaining the variance were evaluated. To test the calibration of the logistic prediction models, Spiegelhalter’s z test was used [54, 55].

Next to regression analyses and in parallel to the procedure described for discriminative ability, areas under the curves (AUC) with 95% confidence intervals (CI) were calculated for STarT-MSK_G_ predicting dichotomised t2-outcomes (dichotomisation see discriminative ability). Moreover, to enable comparison, AUCs were calculated for OMPQ predicting dichotomised t2-outcomes.

Terwee et al. suggested a sample size of 50 patients for construct validity and reliability [45]. Therefore, to enable analyses for subgroups defined by diagnosis, while allowing a drop-out of 10% and considering an uneven distribution (estimated smallest subgroup with 20%), it was aimed to recruit 300 patients in total.

As significance level alpha = 5% was set. Analyses were performed using SPSS version 27.0 and R language and environment for statistical computing, version 4.0.0 [56].

## Results

Consent for participation was given by 287 patients. The mean age was 47 (SD 15.8) years, and 51% were female, with overall 48.8% at low, 43.6% at medium and 7.7% at high risk. (Table 1). Non-consenters (n = 36) on average were 8.6 years (CI 95% 3.7, 13.5), they were older and more often female (66%). During the previous twelve weeks before t0, 64 patients (22.3%) reported having taken some sick leave. The t1 questionnaire was returned by 261 patients (91%), the t2 questionnaire by 246 patients (86%). Forty-five patients (16%) answered the questionnaires before the first contact with the therapist.The median number of contacts before answering the questions of the other 242 patients was 3 (IQR = 3).

**Table 1:** Characteristics of the study population.

	Total			Lower back			Neck			Shoulder			Hip or Knee		
	Mean	SD	n	Mean	SD	n	Mean	SD	n	Mean	SD	n	Mean	SD	n
Age	47.0	15.8	287	46.4	16.0	122	42.5	14.9	65	51.0	13.3	40	50.6	16.6	60
Average pain	4.5	1.8	287	4.6	1.8	122	4.4	1.7	65	4.4	1.9	40	4.4	1.9	60
STarT-MSK_G score	4.7	2.6	287	5.2	2.5	122	4.5	2.7	65	3.6	2.3	40	4.7	2.6	60
OMPQ score	16.4	8.0	286	17.4	8.4	121	16.4	8.1	65	12.2	6.0	40	17.0	7.2	60

		Total		Lower back		Neck		Shoulder		Hip or Knee	
		n	%	n	%	n	%	n	%	n	%
Gender	male	140	48.8%	60	49.2%	28	43.1%	22	55.0%	30	50.0%
	female	147	51.2%	62	50.8%	37	56.9%	18	45.0%	30	50.0%
Education	Professional qualification	43	15.2%	18	15.0%	8	12.5%	7	17.5%	10	16.9%
	Other	12	4.2%	4	3.3%	3	4.7%	2	5.0%	3	5.1%
	General education or lower	62	21.9%	31	25.8%	11	17.2%	6	15.0%	14	23.7%
	Higher education entrance qualification or higher	166	58.7%	67	55.8%	42	65.6%	25	62.5%	32	54.2%
Occupation	20h per week or more	162	57.2%	70	58.3%	40	62.5%	23	57.5%	29	49.2%
	Up to 20h per week	51	18.0%	22	18.3%	9	14.1%	9	22.5%	11	18.6%
	None, unemployed	7	2.5%	3	2.5%	3	4.7%	0	0.0%	1	1.7%
	None, retired	43	15.2%	15	12.5%	6	9.4%	7	17.5%	15	25.4%
	None, househusband or -wife	8	2.8%	3	2.5%	3	4.7%	0	0.0%	2	3.4%
	None of the previous	12	4.2%	7	5.8%	3	4.7%	1	2.5%	1	1.7%
General health	Excellent	6	2.1%	4	3.3%	1	1.5%	1	2.5%	0	0.0%
	Very good	50	17.5%	15	12.4%	10	15.4%	9	22.5%	16	26.7%
	Good	141	49.3%	58	47.9%	41	63.1%	20	50.0%	22	36.7%
	Fair	79	27.6%	37	30.6%	11	16.9%	10	25.0%	21	35.0%
	Poor	10	3.5%	7	5.8%	2	3.1%	0	0.0%	1	1.7%
Sickness certificate	No	223	77.7%	92	75.4%	42	64.6%	36	90.0%	53	88.3%
	Yes	64	22.3%	30	24.6%	23	35.4%	4	10.0%	7	11.7%
Duration of complaints	Up to 11 weeks	98	34.1%	41	33.6%	24	36.9%	10	25.0%	23	38.3%
	12 or more weeks	189	65.9%	81	66.4%	41	63.1%	30	75.0%	37	61.7%
MSK comorbidity	No	170	59.2%	78	63.9%	28	43.1%	23	57.5%	41	68.3%
	Yes	117	40.8%	44	36.1%	37	56.9%	17	42.5%	19	31.7%
STarT-MSK Risk-Group	Low	140	48.8%	50	41.0%	34	52.3%	27	67.5%	29	48.3%
	Medium	125	43.6%	60	49.2%	25	38.5%	12	30.0%	28	46.7%
	High	22	7.7%	12	9.8%	6	9.2%	1	2.5%	3	5.0%

OMPQ: Örebro Musculoskeletal Pain Questionnaire, STarT-MSK_G: STarT-MSK-Tool, German version.

There were 122 patients with lower back complaints, 65 with neck, 40 with shoulder and 60 with hip/knee complaints. Thirty-six patients (12.5%) previously received surgery in the region of their complaints. Additional details on the characteristics of the study population are given in Table 1.

The median time interval between t0 and t1 was 7 (IQR = 8) days and between t0 and t2 181 (IQR = 11) days. The follow-up questionnaires sent to the patients at t1 and t2 were returned by 261 (91%) and 246 (86%) of the participants, respectively. Non-responders at t1 on average were 6.5 years younger than responders, with a large confidence interval (CI 95% -0.7, 13.8) and were less often female (responders 53% female, non-responders 38%). Non-responders at t2 on average were 6.6 years younger than responders, with a confidence interval not including zero (CI 95% 0.9, 12.3) and were less often female (responders 52% female, non-responders 46%).

### Test-retest-reliability

The ICC (t0 to t1) for the STarT-MSK_G_ was 0.75 (95% CI 0.69; 0.81) and therefore, is ‘good’. For individual items the median κ was 0.58 (range 0.42 (item 9) to 0.72 (item 7 and 10)) (Table 2).

**Table 2:** Kappa coefficients of single item test-retest of the STarT-MSK_G_.

	Kappa	95% CI	
		lower	upper
Item 1*	0.58	0.49	0.66
**Item 2**	0.53	0.42	0.63
**Item 3**	0.46	0.37	0.57
**Item 4**	0.63	0.51	0.72
**Item 5**	0.58	0.48	0.65
**Item 6**	0.53	0.44	0.63
**Item 7**	0.72	0.64	0.81
**Item 8**	0.64	0.54	0.75
**Item 9**	0.42	0.27	0.57
**Item 10**	0.72	0.61	0.78

CI: confidence interval

*: squared weights.

### Construct validity

**Fig 1:**
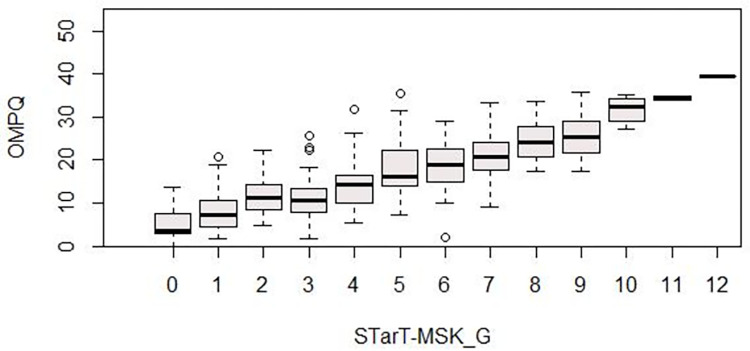
Relation between STarT-MSK_G_ and OMPQ. OMPQ: Örebro Musculoskeletal Pain Questionnaire, STarT-MSK_G: STarT-MSK-Tool, German version.

Correlations for the STarT-MSK_G_-Tool against the OMPQ-Tool was r_s_ = 0.74 (95% CI 0.68, 0.79; convergent construct validity). A visual presentation of the correlation is given in Fig 1. Correlation for the STarT-MSK_G_-Tool against the disability measures consistently was lower, ranging from r_s_ = 0.44 to r_s_ = 0.71 (details displayed in Table 3).

**Table 3:** Correlations STarT-MSK_G_ against disability measures.

Reference	r_s_	95% CI	
		Lower	Upper
**RMDQ**	0.589**	0.46	0.69
**NDI**	0.710**	0.57	0.81
**SPADI_DIS**	0.443**	0.12	0.67
**WOMAC_DIS**	0.536**	0.31	0.74

r_S_: Spearman correlation coefficient; CI: confidence interval; RMDQ: Roland Morris Disability Questionnaire; NDI: Neck Disability Index; SPADI_DIS: Shoulder Pain and Disability Index, subscale disability; WOMAC_DIS: Western Ontario and McMaster Universities Osteoarthritis Index, subscale disability.

** significant correlation.

### Floor/ceiling effects

With 3.8% of patients having a STarT-MSK score of 0 points and 0.3% with the maximal score of twelve points, no floor or ceiling effects were observed.

### Discriminative ability

**Fig 2:**
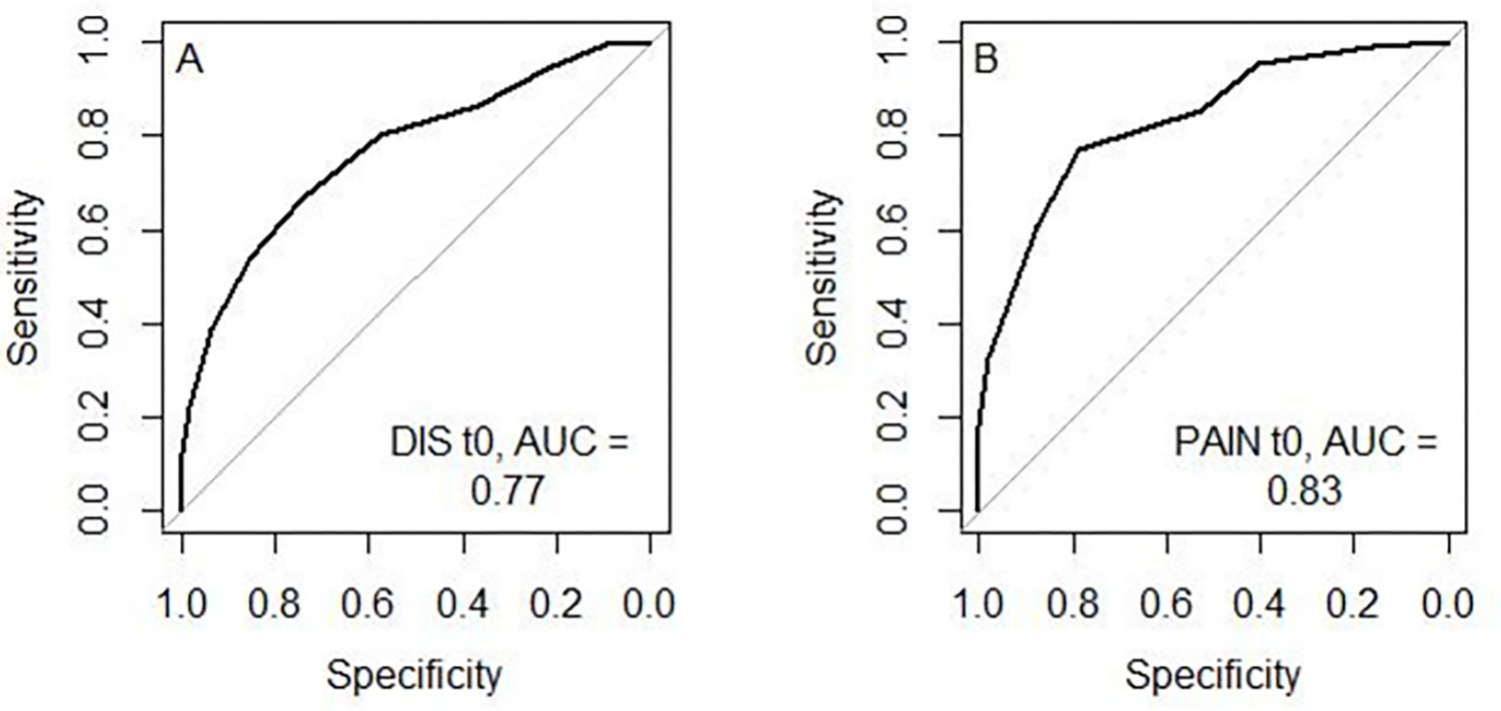
Receiver operating characteristic curves disability and pain at inclusion (t0). Combined disability score (DIS) and pain versus STarT-MSK_G_.

The AUC for STarT-MSK_G_ ability to discriminate disability cases at initial contact was 0.77 (95% CI 0.72, 0.83), indicating ‘acceptable’ discrimination. The AUC for pain was 0.83 (95 CI 0.78, 0.89), indicating ‘good’ discrimination (Fig 2). The AUCs for the different subgroups ranged from 0.68 to 0.85 (Table 4 and S1 Fig).

**Table 4:** Areas under the curve (AUC) by subgroup at initial assessment.

Subgroup	AUC	95% CI	
		Lower	Upper
**Lower back**	0.77	0.68	0.85
**Neck**	0.85	0.76	0.94
**Shoulder**	0.68	0.50	0.86
**Hip or knee**	0.76	0.63	0.88

CI: confidence interval; AUC: area under the curve.

### Predictive ability

#### Regression analyses

The univariate **linear** regression models statistical significance was seen with both p < 0.001, resulting in an amount of explained variance by the STarT-MSK_G_ of 21% (variance explained by OMPQ 17%) for pain at 6 months and 20% (variance explained by OMPQ 19%) for combined disability as shown by adjusted R^2^. The explained variance therefore fell short of the 30% aimed for, both for predicting pain and disability.

For the univariate **logistic** regression models, comparable were statistically significant, with both p < 0.001, resulting in an amount of variance explained by the STarT-MSK_G_ of 29% for pain at 6 months and 26% for combined disability as shown by Nagelkerke’s R^2^. With z = -0.01 for disability and z = 0.07 for pain, Spiegelhalter’s z was non-significant (p = 0.99; p = 0.95).

### Areas under the curves

**Fig 3:**
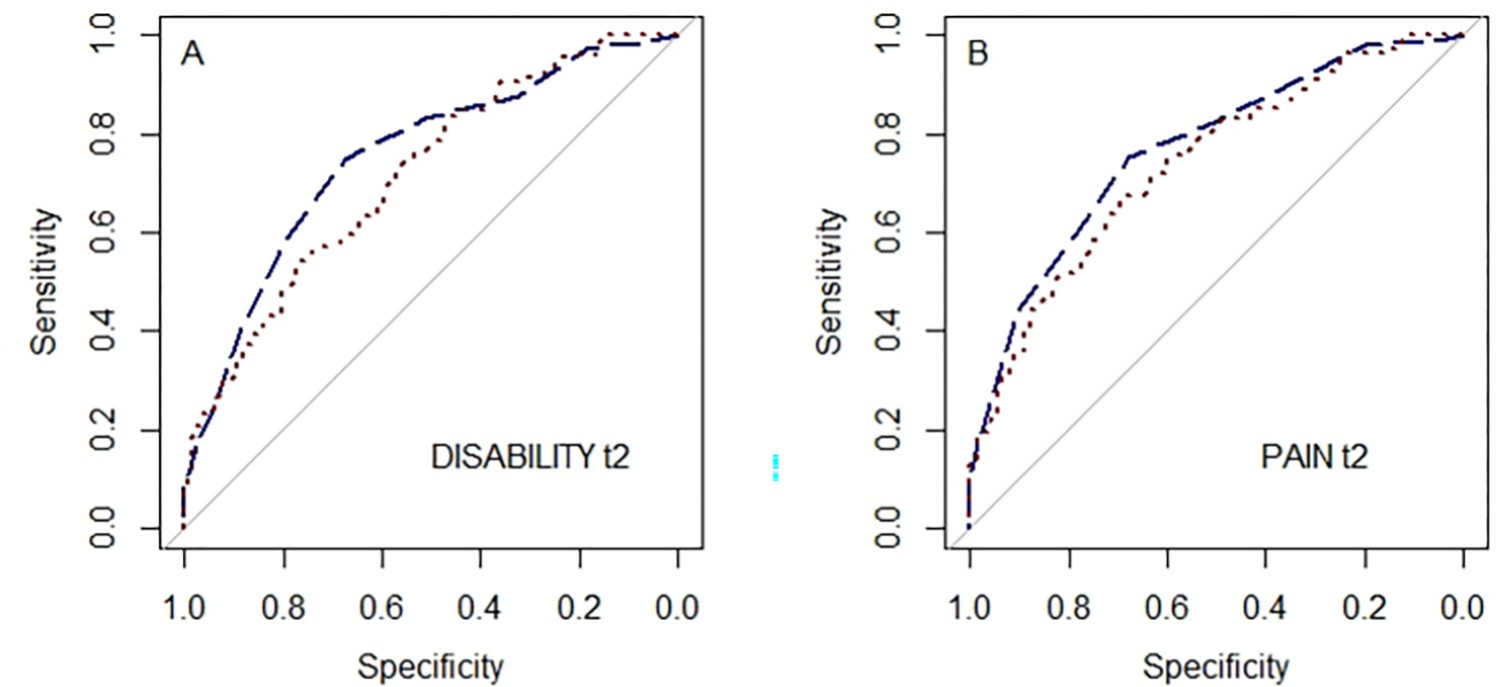
Receiver operating characteristic curves disability and pain at follow-up (t2). Combined DISABILITY score and PAIN versus STarT-MSK_G_ (dashed) and Örebro Musculoskeletal Pain Questionnaire (dotted).

The AUC for STarT-MSK_G_ ability to predict pain-cases at follow-up was 0.77 (95 CI 0.71, 0.83). The AUC for disability was 0.76 (95% CI 0.70, 0.82), indicating ‘acceptable’ prediction (Fig 3). The AUC for OMPQ predicting pain-cases at follow-up was 0.74 (95% CI 0.68, 0.80) and for disability-cases 0.72 (95% CI 0.65, 0.78) (Fig 3). The AUCs for disability by subgroup ranged from 0.70 to 0.88 (Table 5 and S2 Fig), indicating overall ‘acceptable’ discrimination and ‘good’ discrimination for patients with hip or knee complaints.

**Table 5:** Areas under the curve (AUC) by subgroup at follow-up.

Subgroup	AUC	95% CI	
		Lower	Upper
**Lower back**	0.70	0.60	0.79
**Neck**	0.76	0.63	0.89
**Shoulder**	0.75	0.58	0.94
**Hip or knee**	0.88	0.78	0.98

CI: confidence interval; AUC: area under the curve.

## Discussion

After cross-cultural adaptation of the STarT-MSK, a German version is now available and first information on its psychometric properties was established. Overall, these are promising, especially with good test-retest reliability and good construct validity. The instrument explained an amount of variance six months after the first measurement with slightly stronger preditive values than those for the OMPQ. Nevertheless, it fell short of the ≥30% target.

To test the construct validity, the Örebro Musculoskeletal Pain Questionnaire (OMPQ) was used [35]. The good correlation between the instruments confirm that the STarT-MSK_G_ assesses risk for persisting pain disability. In comparison, correlations with instruments measuring disability was lower.

Predictive properties checked by ROC-Analyses resulted in acceptable AUCs that were higher than those of the OMPQ, although with extensively overlapping CI. The calculated, logistic and linear models with pain or disability outcomes explained from 20% to 29% of the variance in outcome, but did not exceed the pre-specified target of R^2^>30%. The number of 30% was estimated based on results from the development study, being unpublished at the time [15]. The achieved amount of explained variance fits very well to that from the external validation for the original version of the STarT-MSK [15]. In future studies the added value of the STarT-MSK_G_ together with covariables could be analysed for example in multiple regression analyses. Comparably, for the German version of the STarT-Back-Tool, adding a one item-variable capturing global health status and the baseline score of the outcome (disability) successfully increased the variance explained to ≥45% [10]. Moreover, the suggestions given by Beneciuk et al. to use change-scores of the STarT-Tool might be considered [57]. In the present study the latter was not done, since the aim of data collection at t1 was to test retest-reliability. Van den Broek et al. just recently examined the predictive validity of the Dutch version of the STarT-MSK, by choosing a different statistical method [17]. Calculating relative risks, they showed that patients at low risk had a better prognosis than those at medium and especially than those at high risk. Major differences to that work–next to the language–are a shorter follow-up and a much smaller sample size leading to a total of three high-risk patients.

The treatment was not influenced by the researchers in the presented study, thus, it can be assumed that the content will have influenced the outcome at follow-up and the variance explained by the regression analyses. An alternative would have been to standardize the procedures, but such a shift from an observational to an experimental design would have led to costs exceeding the available resources for this project. On the other hand, to withhold therapy would have been unethical. Considering the other positive properties established for reliability and validity of the STarT-MSK_G_, it would be worthwhile to develop a study design specifically aiming at improving prediction.

All predictive ROC at least resulted in acceptable AUC. The cut-offs used to differ between cases and non-cases were derived from the literature [47–50]. Nonetheless, various methods exist to define cut-offs leading to different values [58]. A choice of different cut-offs might have resulted in diverging AUC.

The development of the STarT-MSK_G_ is related to that of the STarT-Back-Tool. While the area of application of STarT-Back-Tool is limited to low back pain [8], a strength of the STarT-MSK_G_ is its appropriability for patients with a variety of musculoskeletal complaints. In practice, such a possibility for generic use makes clinical processes easier with one instrument fitting for a broader group of patients. The administrative burden can be reduced, since often patients present with complaints at several sites simultaneously [59, 60]. Moreover, in future it might enable comparison of different patient-subgroups [61, 62].

Another strength of the STarT-MSK is that matched treatments were compiled and instruments assisting clinicians in decision-making are under development [24, 28, 63]. Such instruments help the clinician to address the patients’ needs more specifically, eg. choosing cognitive behavioural-based approaches for patients at high risk of an unfavorable outcome [64, 65]. The knowledge of the measurement properties of the STarT-MSK_G_ strengthens its use for this purpose. Since practitioners have mixed ideas about how to best make use of prognostic tools [66, 67], strategies on how to best implement them should be further developed and detailed studies to describe the added value should be conducted [6].

### Strength and weaknesses

Reference for the development of the STarT-MSK at Keele (UK) was the STarT-Back-Tool [8, 18]. Three of the authors (SK, RH, JCH) were involved in translation of the latter to German and testing of its psychometric properties, resulting in a valid version [32, 68]. The knowledge derived from this process, was an advantage for the work on STarT-MSK, since the researchers were familiar with the underlying concept.

Next to the German version multiple other translations were done including the Dutch, French, Hebrew and Norwegian versions which were validated. However, three of those studies were conducted with smaller sample-sizes and only one with a design enabling determination of the instrument’s predictive ability [17, 19, 21]. For the Dutch version the predictive ability was confirmed, although the cited authors suggested a further external validation study [17].

The low amount of dropout in this study is an area of strength. It is substantially lower than the benchmark set by the Cochrane Back and Neck Group for long term follow-up [69]. This indicates that the developed strategy for data collection worked well, which is in line with descriptions on the low burden of online data collection [70].

Concerning the sample size, in the literature minimal numbers of 50 to 100 participants are required for validation [45, 71]. In total this was easily met and the lower number was also met for the different pain sites except for patients with shoulder complaints. Results for this subgroup should be considered preliminary and confirmed in future works, probably in a specifically tailored study, since these patients are most difficult to recruit [60]. The number of included high-risk patients, who are often seldom, even was comparably high, especially when considering the physiotherapeutic setting [17, 72].

## Conclusion

The German version of the STarT-MSK-Tool is a valid instrument for use across multiple musculoskeletal conditions and is availabe for use in clinical practice. It fulfils the fundamental requirements for an assessment instrument having shown good test-retest-reliability, face, construct validity and predictive validity when analysing ROC-Curves. The instrument explains a considerable amount of variance in six month pain and disability scores. However, whilst the prognostic abilities are comparable to those of the existing reference standard (OMPQ), as the variance was lower than the target set a priori, it is recommended that future research should seek to raise the predictive abilities of this tool further.

## Supporting information

S1 Fig Receiver operating characteristic curves disability by subgroup. Disability-scores versus STarT-MSK_G_ total score; RMDQ: Roland Morris Disability Questionnaire; NDI: Neck Disability Index; SPADI: Shoulder Pain and Disability Index, subscale disability; WOMAC: Western Ontario and McMaster Universities Osteoarthritis Index, subscale disability; t0: initial.
(TIF)

S2 Fig Receiver operating characteristic curves disability by subgroup at follow-up. Disability-scores versus STarT-MSK_G_ total score; RMDQ: Roland Morris Disability Questionnaire; NDI: Neck Disability Index; SPADI: Shoulder Pain and Disability Index, subscale disability; WOMAC: Western Ontario and McMaster Universities Osteoarthritis Index, subscale disability; t2: follow-up.
(TIF)
