# Supplementary figures and images for: Validation of the German version of the STarT-MSK-Tool: A cohort study with patients from physiotherapy clinics
Source: PLoS One. 2022 Jul 1;17(7):e0269694. doi: 10.1371/journal.pone.0269694 (PMC9249194; doi:10.1371/journal.pone.0269694)

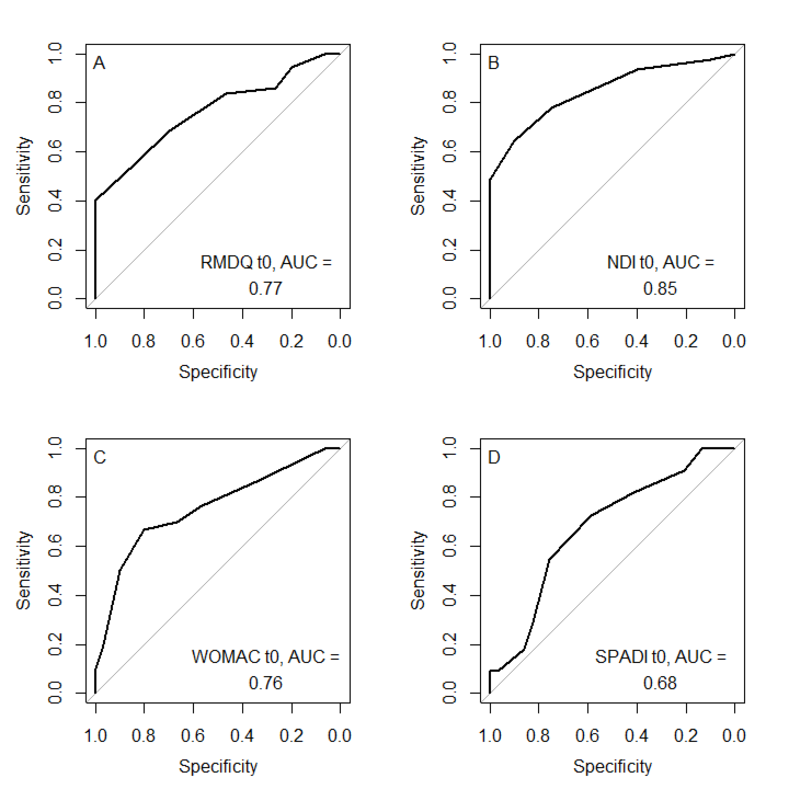

Supplement: S1 Fig — Disability-scores versus STarT-MSKG total score; RMDQ: Roland Morris Disability Questionnaire; NDI: Neck Disability Index; SPADI: Shoulder Pain and Disability Index, subscale disability; WOMAC: Western Ontario and McMaster Universities Osteoarthritis Index, subscale disability; t0: initial. (TIF) [file pone.0269694.s001.tif]

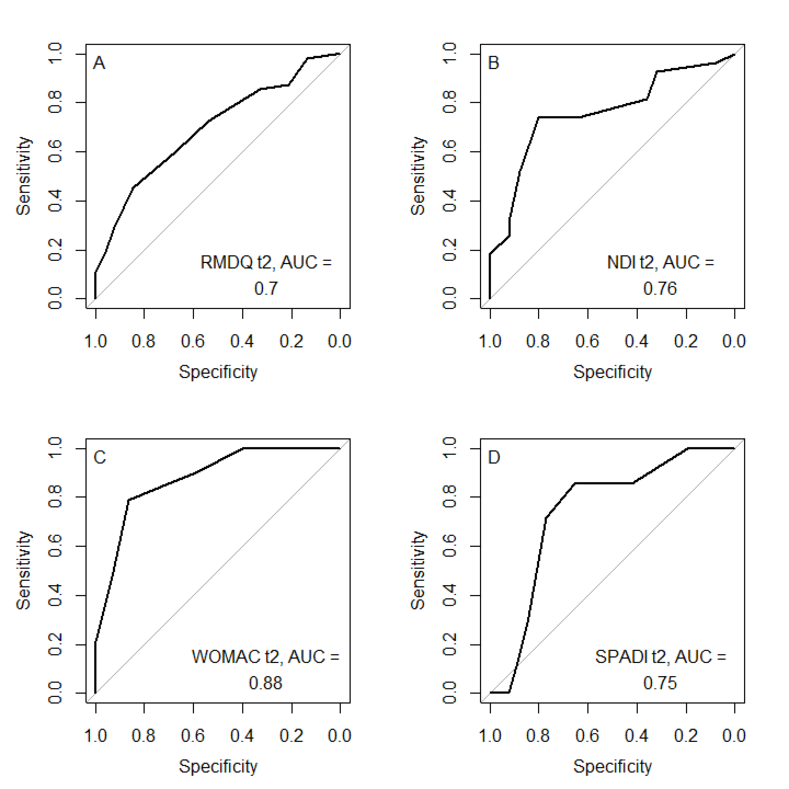

Supplement: S2 Fig — Disability-scores versus STarT-MSKG total score; RMDQ: Roland Morris Disability Questionnaire; NDI: Neck Disability Index; SPADI: Shoulder Pain and Disability Index, subscale disability; WOMAC: Western Ontario and McMaster Universities Osteoarthritis Index, subscale disability; t2: follow-up. (TIF) [file pone.0269694.s002.tif]
